# A Japanese national survey on IMRT/SBRT in 2023 by the JASTRO High-Precision External Beam Radiotherapy Group

**DOI:** 10.1093/jrr/rraf009

**Published:** 2025-04-16

**Authors:** Masahide Saito, Shuichi Ozawa, Takafumi Komiyama, Masaki Kokubo, Yoshiyuki Shioyama, Yukinori Matsuo, Takashi Mizowaki, Tomoki Kimura, Hideyuki Harada, Hiroshi Igaki, Naoki Tohyama, Masahiko Kurooka, Mitsuhiro Nakamura, Yu Kumazaki, Hidekazu Suzuki, Hikaru Nemoto, Nagata Yasushi, Hiroshi Onishi

**Affiliations:** Department of Radiology, University of Yamanashi, 1110 Shimokato, Chuo-city, Yamanashi, Yamanashi 409-3898, Japan; Division of Medical Physics, Hiroshima High-Precision Radiotherapy Cancer Center, 3-2-2, Futabanosato, Higashiku, Hiroshima, Hiroshima 732-0057, Japan; Department of Radiology, University of Yamanashi, 1110 Shimokato, Chuo-city, Yamanashi, Yamanashi 409-3898, Japan; Department of Radiation Oncology, Kobe City Medical Center General Hospital, 2-1-1, Minatojima-Minamimachi, Chuo, Kobe, Hyogo 650-0047, Japan; Division of Radiation Oncology, Ion Beam Therapy Center, SAGA-HIMAT Foundation, 3049, Harakoga-machi, Tosu, Saga 841-0071, Japan; Department of Radiation Oncology, Kindai University Faculty of Medicine, 377-2 Ono-higashi, Osaka-sayama, Osaka 589-8511, Japan; Department of Radiation Oncology and Image-Applied Therapy, Graduate School of Medicine, Kyoto University, 54 Shogoinkawahara-cho, Sakyo-ku, Kyoto 606-8507, Japan; Department of Radiation Oncology Kochi Medical School, Kochi University, Kohasu, Okou Town, Nankoku City, Kochi 783-8505, Japan; Division of Radiation Therapy, Shizuoka Cancer Center Hospital, 1007 shimonagakubo, nagaizumi-cho, sunto-gun, Shizuoka 411-8777, Japan; Department of Radiation Oncology, National Cancer Center Hospital, 5-1-1 Tsukiji, Chuo-ku, Tokyo 104-0045, Japan; Department of Radiological Sciences, Komazawa University, 1-23-1 Komazawa, Setagaya-Ku, Tokyo 154-8525, Japan; Department of Radiation Therapy, Tokyo Medical University Hospital, 6-7-1 Nishishinjuku, Shinjuku-ku, Tokyo 160-0023, Japan; Department of Advanced Medical Physics, Graduate School of Medicine, Kyoto University, 53 Shogoinkawahara-cho, Sakyo-ku, Kyoto 606-8507, Japan; Department of Radiation Oncology, International Medical Center, Saitama Medical University, 1397-1 Yamane, Hidaka, Saitama 350-1298, Japan; Department of Radiological Technology, Faculty of Medical Technology, Teikyo University, 2-11-1 Kaga, Itabashi-Ku, Tokyo 173-8605, Japan; Department of Radiology, University of Yamanashi, 1110 Shimokato, Chuo-city, Yamanashi, Yamanashi 409-3898, Japan; Department of Radiation Oncology, Chugoku Rosai Hospital, Hirotagaya 1-5-1, Kure, Hiroshima 737-0193, Japan; Department of Radiology, University of Yamanashi, 1110 Shimokato, Chuo-city, Yamanashi, Yamanashi 409-3898, Japan

**Keywords:** IMRT, SBRT, IGRT, respiratory motion management

## Abstract

The purpose of this study was to investigate the utilization and implementation of stereotactic body radiotherapy (SBRT) and intensity-modulated radiotherapy (IMRT) in Japan up to 2023. The survey was conducted by the Japanese Society for Radiation Oncology High-Precision External Beam Radiotherapy Group Subcommittee from December 2023 to February 2024. The study targeted patients treated with IMRT or SBRT between January 2021 and December 2022. A comprehensive web-based questionnaire was distributed to 880 facilities, with separate sections for radiation oncologists and medical physicists/radiotherapy technologists. A total of 360 facilities responded (response rate: 40.9%) for the section of radiation oncologists, and 405 facilities responded (response rate: 46.0%) for medical physicists/radiotherapy technologists, providing data on the implementation status, techniques, workload and challenges associated with IMRT and SBRT. Based on the responses in the section of radiation oncologists, IMRT was used in 68.6% of responding institutes, and SBRT in 87.8%. VMAT emerged as the most common IMRT technique (78.3%). The survey highlighted a high demand for medical physicists to perform IMRT (86.9%). Based on the responses in the section of medical physicists/radiotherapy technologists, 84.6% of the facilities that have not performed IMRT reported that the main reason was a lack of radiation oncologists. Furthermore, the survey also noted significant variations in prescribed doses and margin sizes across facilities, indicating the need for further standardization. High-precision radiation techniques such as IMRT and SBRT are getting popular, however, the facility requirements which mandate the presence of at least two radiation oncologists prevents IMRT from becoming more widespread in Japan.

## INTRODUCTION

Stereotactic body radiotherapy (SBRT) and intensity-modulated radiotherapy (IMRT) have become essential techniques in contemporary radiation therapy. In Japan, SBRT was incorporated into the national health insurance system in 2004 to treat lung and liver cancers. Particularly for lung cancer, due to its outstanding therapeutic outcomes [1, 2], and SBRT is now almost routinely provided at leading medical centers across the country. Subsequently, the coverage of SBRT under the national health insurance system was extended to include prostate cancer in 2016 and further expanded in 2020 to treat spinal metastases and oligometastases with up to five lesions.

Regarding to IMRT, it became an advanced medical treatment in 2006, and was first covered by the national health insurance system for localized solid tumors in 2008. In recent years, many types of treatment machines have been developed and introduced, and IMRT is becoming increasingly available as a standard option. In addition, the workload required for external irradiation has significantly changed since the introduction of IMRT. Recently, a survey on this workload showed that IMRT irradiation can now be performed in approximately the same amount of time as 3-dimensional conformal radiotherapy (3DCRT) irradiation [3]. On the other hand, several issues must be addressed for the widespread adoption of IMRT in Japan, including the consolidation and standardization of facilities, and the education or training of staff familiar with treatment planning [4].

In Japan, Japanese Society for Radiation Oncology (JASTRO) structural surveys and Japanese Radiation Oncology Database (JROD) surveys have been conducted continuously [5–10]. However, this is a comprehensive survey of all radiation therapies, and a more in-depth survey is needed to investigate various issues specific to high-precision radiotherapy such as IMRT and SBRT. No survey has been conducted on the number of cases, various techniques or irradiation methods specific to high-precision treatments such as IMRT and SBRT, nor has there been any survey on the current challenges and requests regarding the national health insurance system for such high-precision irradiation. Given the increasing complexity of high-precision irradiation work, a more specialized survey focused on high-precision external irradiation is needed, distinct from the JASTRO structural survey. Previously, the actual utilization of high-precision radiotherapy in Japan was first surveyed in the 2000s, which was SBRT, and surveys have been conducted continuously since then [11–13]. In this study, a survey on the utilization of IMRT/SBRT up to 2023 was carried out by the JASTRO High-Precision External Beam Radiotherapy Group Subcommittee, aimed at assessing the current implementation of IMRT and SBRT in Japan. IMRT in this study refers to all irradiation techniques, including Static MLC (step and shoot), Dynamic MLC (sliding window), VMAT, Dynamic Wave Arc and Helical irradiation. SBRT, on the other hand, refers to all irradiation techniques targeting extracranial sites within the body, using all irradiation devices except the Gamma Knife.

## MATERIAL AND METHODS

### Overview of web-based questionnaire

The survey encompassed 880 facilities equipped with external beam radiotherapy systems, including high-energy X-ray equipment, as of December 2023. The survey period was from 20 December 2023 to 13 February 2024. Eligible patients were those treated with IMRT or SBRT between 1 January 2021 and 31 December 2022. The survey questionnaire items were independently designed by two working groups: one comprising radiation oncologists and the other consisting of medical physicists and radiation technologists. The radiation oncologists’ section included case studies, resulting in a smaller number of questions focused on topics such as IMRT and SBRT implementation and clinical procedures. In contrast, the medical physicist/radiation technologist section featured more questions, addressing technical aspects not covered in the physicians’ section. The web-based questionnaire system was constructed by RTQM system Inc. (Hiroshima, Japan). The URL for the web system was distributed to the target facilities via postal mail, email through the JASTRO mailing list, and the JASTRO website.

### Questions for the radiation oncologist section

A total of 27 questions were prepared as disclosable items. The content included questions about basic information on the facility, IMRT implementation status (including IGRT), online adaptive radiotherapy (online-ART) implementation status, SBRT implementation status (including respiratory motion management techniques), radiation therapy procedures and the cumulative number of treated cases. Details of the questions are shown in the results section and table.

### Questions for the medical physicist/radiation technologist section

A total of 61 questions were prepared as disclosable items. The content included questions on basic information about the facility and staff, IMRT and SBRT implementation status, conditions in the radiotherapy room, radiotherapy planning equipment, treatment planning, dose verification, other tasks and near misses. Details of the questions are shown in the results section and each table.

## RESULTS

### Radiation oncologist section—summary and general information for this survey

Table 1 shows the results of the questionnaire in the radiation oncologist section. Of the 880 facilities surveyed, 360 responded (response rate: 40.9%).

**Table 1:** Survey result of the questionnaire in the radiation oncologist section

No.	Contents	*n*	Answer
1.1	Would you like to use remote-radiotherapy treatment planning assistance for IMRT?	360	Yes: 127 (35.3%) Somewhat negative: 99 (27.5%) No: 104 (28.9%) Unknown: 30 (8.3%)
1.2	Do you think a medical physicist is necessary to perform IMRT?	360	Yes: 313 (86.9%) Neither agree nor disagree: 43 (11.9%) No: 4 (1.1%)
1.3	Do you have plans to increase the number of patients treated with IMRT in the future?	281	Increase: 191 (68%) Maintenance of the status quo: 90 (32%)
1.4	What percentage of all radiotherapy treatments are performed with IMRT at your institution?	281	75–100%: 27 (9.6%) 50–75%: 54 (19.2%) 20–50%: 154 (54.8%) 10–20%: 35 (12.5%) 5–10%: 10 (3.6%) 1–5%: 1 (0.4%)
1.5	IMRT techniques used in your institute (duplicate).	281	Static MLC (step and shoot): 32 (11.4%) Dynamic MLC (sliding window): 59 (21.0%) VMAT: 220 (78.3%) Dynamic wave arc: 5 (1.8%) Helical irradiation: 57 (20.3%) Others: 6 (2.1%)
1.6	Do the radiotherapy technologists in your institution perform primary image matching for IGRT for IMRT?	277	Yes: 267 (96.4%) No: 10 (3.6%)
1.7	If you answered no to the above question, please explain why not (Duplicate)	10	No established education and training system: 4 (40%) Inability to share information about IGRT well: 2 (20%) Because the radiotherapy technologists have not created treatment plans: 4 (40%) Others: 3 (30%)
1.8	Reasons for not implementing IMRT (Duplicate)	75	Due to one full-time radiation oncologist: 45 (60%) IMRT system is not fully equipped: 26 (34.7%) Shortage of medical physicist and radiotherapy technologist: 16 (21.3%) No full-time radiation oncologist: 7 (9.3%) Busy with other duties: 4 (5.3%) Not contributing to hospital management: 1 (1.3%) No reliable IMRT data: 8 (10.7%) Currently under consideration: 18 (24%) Others: 13 (17.3%)
1.9	Do you provide on-line ART*?	360	Yes: 15 (4.2%) No: 345 (95.8%)
1.10	If performed, which images are used for implementation? (Duplicate)	15	CT images (including CBCT images): 11 (73.3%) MR Image: 7 (46.7%)
1.11	Does your institution currently perform SBRT?	360	Yes: 316 (87.8%) No: 44 (12.2%)
1.12	Do you think a medical physicist is necessary to perform SBRT?	360	Yes: 283 (78.6%) Neither agree nor disagree: 71 (19.7%) No: 6 (1.7%)
1.13	Is respiratory motion management performed during SBRT?	311	Yes: 295 (94.9%) No: 16 (5.1%)
1.14	Organs for performing respiratory motion management during SBRT (Duplicate)	304	Lung: 295 (97.0%) Liver: 229 (75.3%) Adrenal glands: 101 (33.2%) Kidney: 73 (24.0%) Lymph nodes: 59 (19.4%) Pancreas: 50 (16.4%) Bile duct: 33 (10.9%) Prostate: 4 (1.3%) Left mamma: 6 (2%) Right mamma: 3 (1%) Other: 6 (2%) Not carried out: 8 (2.6%)
1.15	Is respiratory motion management performed apart from SBRT (duplicate)?	313	IMRT/VMAT: 183 (58.5%) 3DCRT: 194 (62.0%) Not carried out: 63 (20.1%)
1.16	Organs for performing respiratory motion management except SBRT (duplicate)	290	Lung: 213 (73.4%) Liver: 157 (54.1%) Left mamma: 113 (39.0%) Pancreas: 110 (37.9%) Adrenal glands: 96 (33.1%) Kidney: 79 (27.2%) Lymph nodes: 68 (23.4%) Bile duct: 67 (23.1%) Esophagus: 64 (22.1%) Right mamma: 13 (4.5%) Prostate: 2 (0.7%) Other (description): 22 (7.6%) Not carried out: 42 (14.5%)
1.17	What is the maximum number of lesions where SBRT is performed for oligo-metastases?	293	1 lesion: 16 (5.5%) 2 lesions: 38 (13%) 3 lesions: 136 (46.4%) 4 lesions: 18 (6.1%) 5 lesions: 82 (28%) 10 lesions: 3 (1%)
1.18	If SBRT is performed for five or fewer oligo metastases, when is the medical fee charged (Duplicate)?	304	Only once at the time of initial lesion treatment: 240 (78.9%) Only once after treatment of all lesions: 15 (4.9%) Case-by-case basis for each lesion: 32 (10.5%) Others: 17 (5.6%)
1.19	Utilization of Space-OAR (duplicate)	360	Used in IMRT (except SBRT): 110 (30.6%) Used in SBRT: 41 (11.4%) Used in other techniques: 37 (10.3%) Not used: 208 (57.8%)
1.20	(If yes,) what is the percentage of cases using Space-OAR (among all prostate cancer cases)?	161	80% or more: 60 (37.3%) 80–60%: 21 (13%) 60–40%: 24 (14.9%) 40–20%: 19 (11.8%) 20% or less: 37 (23%)
1.21	Utilization of PGA spacers (NESKEEP)	360	Used: 14 (3.9%) Not used: 346 (96.1%)
1.22	Utilization of fiducial marker	360	Used in IMRT (except SBRT): 75 (20.8%) Used in SBRT: 98 (27.2%) Used for other techniques: 21 (5.8%) Not used: 213 (59.2%)
1.23	(If yes,) The name of using product for each region	146	Lung: gold anchor 9, VISICOIL 4, gold marker 28, ACCUROC 0, Others 2 Liver: gold anchor 40, VISICOIL 36, gold marker 3, ACCUROC 5, others 3 Prostate: gold anchor 41, VISICOIL 35, gold marker 7, ACCUROC 8, others 3 Other regions: gold anchor 3, VISICOIL 4, gold marker 0, ACCUROC 0, others 1

Abbreviations: IMRT (intensity-modulated radiation therapy), MLC (multi-leaf collimator), VMAT (volumetric modulated arc therapy), IGRT (image-guided radiation therapy), CBCT (cone beam computed tomography), MR (magnetic resonance).

The institutions surveyed included university hospitals (*n* = 70; 19.4%), national hospitals (*n* = 38; 10.6%), public hospitals (*n* = 107; 29.7%), general hospitals (*n* = 123; 34.2%) and other institutions (*n* = 22; 6.1%). In Japan, there were 461 designated cancer care hospital (DCCH)s accredited by the Ministry of Health, Labor and Welfare [14,], and 288 of them responded to the survey (response rate: 62.5%).

The demand for medical physicists was high for IMRT (86.9%) and SBRT (78.6%). Among facilities, 68% planned to increase IMRT use, with 54.8% treating 20–50% of patients with IMRT. SBRT was implemented by 87.8% of respondents, while 60% of the 75 facilities not using IMRT cited having only one full-time radiation oncologist.

### Radiation oncologist section—technical aspects related to IMRT/SBRT

Table 1 highlights technical aspects of IMRT/SBRT. IGRT is crucial for IMRT, with 96.4% of facilities delegating IGRT matching to radiotherapy technologists. Among 10 facilities not shifting IGRT tasks, 40% cited lack of education/training, 40% noted absence of treatment planning by technologists, 20% mentioned poor IGRT communication, and 30% indicated other reasons. VMAT is the dominant IMRT technique (78.3%), while 35.3% are open to remote radiotherapy planning. Only 4.2% perform on-line ART, mainly using CT (73.3%) or MR images (46.7%).

For SBRT, 94.9% employ respiratory motion management, primarily for lung cases (97%). Beyond SBRT, motion management is used in IMRT/VMAT (58.5%), 3DCRT (62%) and rarely elsewhere (20.1%). Most facilities limit SBRT to three oligo-metastatic lesions (46.4%) and charge medical fees once at initial treatment (80.3%). Space-OAR is used by 30.6% for IMRT, 11.4% for SBRT and 57.8% do not use it. Polyglycolic Acid spacer (PGA) spacers are rarely utilized (3.9%), while fiducial markers are employed in IMRT (20.8%), SBRT (27.2%) and other techniques (5.8%), with Gold Anchor and similar markers commonly used.

### Radiation oncologist section—the number of IMRT and SBRT cases

**Fig. 1:**
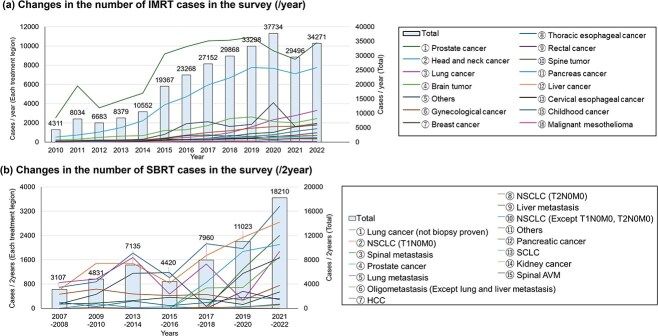
The changes of the number of IMRT and SBRT cases. (A) changes in the number of IMRT cases in the survey (/year), and (B) changes in the number of SBRT cases in the survey (/2 year) is shown. The horizontal axis is the number of years, and the vertical axis shows the number of cases for each case on the left and the total number of cases on the right. The legends of the two figures are ordered by the number of cases in the latest year (2023).

Figure 1 shows the evolution of the number of IMRT and SBRT cases. COVID-19 might reduce the number of IMRT cases, which has since recovered. The most common treatment sites were prostate cancer, head and neck cancer, lung cancer, brain tumors, gynecological cancer, breast cancer, thoracic esophageal cancer and rectal cancer, in that order. The number of SBRT cases was increasing significantly, with the most common treatment sites being lung cancer (not biopsy proven), Non-small cell lung cancer (NSCLC) (T1N0M0), spinal metastasis, prostate cancer and lung metastasis, in that order. Table 2 shows the results of the Top 5 prescribed doses of SBRT for each site and the utilization ratio of prescription methods (volume or isocenter). Various prescribed doses were used for each organ, and most prescription methods were based on the PTV volume rather than the isocenter. Table 3 shows Grade 5 adverse events due to SBRT. 72 cases with Grade 5 were observed by 2022, resulting in an incidence rate of 0.1% when divided by the cumulative number of SBRT cases in this study up to 2022 (56 686 cases). Because a few institutions did not respond in 2022, total number of Grade 5 adverse events decreased.

**Table 2:** The results of the Top 5 prescribed doses of SBRT for each site and utilization ratio for prescription methods (volume or isocenter)

		Top 5 SBRT prescription dose in this survey					Utilization ratio for prescription methods
	*n*	No. 1	No. 2	No. 3	No. 4	No. 5	
NSCLC (T1N0M0) peripheral lesion	276	48Gy/4Fr (PTV D95%) (*n* = 84)	42Gy/4Fr (PTV D95%) (*n* = 44)	50Gy/4Fr (PTV D95%) (*n* = 34)	48Gy/4Fr (PTV DXX%) (*n* = 14)	48Gy/4Fr (isocenter) (n = 14)	Volume: 92.8% Isocenter: 7.2%
NSCLC (T1N0M0) central lesion	275	60Gy/8Fr (PTV D95%) (*n* = 57)	50Gy/8Fr (PTV D95%) (*n* = 18)	60Gy/10Fr (PTV D95%) (*n* = 14)	60Gy/8Fr (PTV DXX%) (*n* = 13)	60Gy/8Fr (isocenter) (*n* = 12)	Volume: 81.1% Isocenter: 18.9%
NSCLC (T2N0M0) peripheral lesion	267	48Gy/4Fr (PTV D95%) (*n* = 49)	42Gy/4Fr (PTV D95%) (*n* = 29)	55Gy/4Fr (PTV D95%) (*n* = 13)	60Gy/8Fr (PTV D95%) (*n* = 11)	50Gy/4Fr (PTV D95%) (*n* = 8)	Volume: 89.5% Isocenter: 10.5%
NSCLC (T2N0M0) central lesion	211	60Gy/8Fr (PTV D95%) (*n* = 43)	50Gy/8Fr (PTV D95%) (*n* = 12)	60Gy/8Fr (isocenter) (n = 12)	60Gy/8Fr (PTV DXX%) (*n* = 9)	60Gy/10Fr (PTV D95%) (*n* = 8)	Volume: 84.4% Isocenter: 15.6%
Lung metastasis	306	48Gy/4Fr (PTV D95%) (*n* = 67)	42Gy/4Fr (PTV D95%) (*n* = 30)	50Gy/4Fr (PTV D95%) (*n* = 22)	55Gy/4Fr (PTV D95%) (*n* = 11)	60Gy/5Fr (PTV D95%) (n = 11)	Volume: 90.8% Isocenter: 9.2%
HCC	231	40Gy/5Fr (PTV D95%) (*n* = 56)	48Gy/4Fr (PTV D95%) (*n* = 17)	40Gy/5Fr (PTV DXX%) (*n* = 15)	50Gy/5Fr (PTV D95%) (*n* = 13)	40Gy/4Fr (PTV D95%) (*n* = 10)	Volume: 89.2% Isocenter: 10.8%
Liver metastasis	212	40Gy/5Fr (PTV D95%) (*n* = 35)	50Gy/5Fr (PTV D95%) (*n* = 22)	48Gy/4Fr (PTV D95%) (*n* = 19)	60Gy/5Fr (PTV D95%) (*n* = 7)	50Gy/5Fr (PTV DXX%) (*n* = 6)	Volume: 89.2% Isocenter: 10.8%
Spinal AVM	7	25Gy/5Fr (PTV D95%) (n = 1)	24Gy/4Fr (PTV D95%) (n = 1)	24Gy/2Fr (PTV DXX%) (n = 1)	21Gy/3Fr (PTV D95%) (n = 1)	20Gy/3Fr (PTV D95%) (n = 1)	Volume: 100% Isocenter: 0%
Prostate cancer	69	36.25Gy/5Fr (PTV D95%) (*n* = 23)	36.25Gy/5Fr (PTV DXX%) (*n* = 4)	35Gy/5Fr (PTV D95%) (n = 4)	37.5Gy/5Fr (PTV D95%) (*n* = 3)	37.25Gy/5Fr (PTV D95%) (n = 3)	Volume: 97.1% Isocenter: 2.9%
Kidney cancer	77	40Gy/5Fr (PTV D95%) (*n* = 13)	70Gy/10Fr (PTV D95%) (*n* = 6)	60Gy/10Fr (PTV D95%) (*n* = 5)	35Gy/5Fr (PTV D95%) (n = 5)	40Gy/4Fr (PTV D95%) (*n* = 2)	Volume: 93.5% Isocenter: 6.5%
Adrenal tumor	89	40Gy/5Fr (PTV D95%) (*n* = 15)	35Gy/5Fr (PTV D95%) (*n* = 13)	35Gy/5Fr (PTV DXX%) (*n* = 7)	50Gy/10Fr (PTV D95%) (*n* = 4)	48Gy/4Fr (PTV D95%) (*n* = 3)	Volume: 94.4% Isocenter: 5.6%
Spinal metastasis	193	35Gy/5Fr (PTV D95%) (*n* = 44)	24Gy/2Fr (PTV D95%) (*n* = 19)	24Gy/2Fr (PTV DXX%) (*n* = 8)	30Gy/5Fr (PTV D95%) (n = 8)	35Gy/5Fr (PTV DXX%) (n = 8)	Volume: 94.8% Isocenter: 5.2%
Lymph node metastasis	107	35Gy/5Fr (PTV D95%) (*n* = 32)	40Gy/5Fr (PTV D95%) (*n* = 11)	35Gy/5Fr (PTV DXX%) (*n* = 6)	30Gy/5Fr (PTV D95%) (*n* = 5)	60Gy/10Fr (PTV D95%) (*n* = 4)	Volume: 97.2% Isocenter: 2.8%
Pancreatic cancer	50	40Gy/5Fr (PTV D95%) (n = 5)	35Gy/5Fr (PTV D95%) (n = 5)	50Gy/5Fr (PTV D95%) (*n* = 2)	50Gy/10Fr (PTV D95%) (n = 2)	45Gy/15Fr (PTV D95%) (n = 2)	Volume: 100% Isocenter: 0%

Abbreviations: D95% (dose received by 95% of the target volume), DXX% (dose received by XX% of the target volume), HCC (hepatocellular carcinoma), AVM (arteriovenous malformation).

**Table 3:** The accumulated number of Grade 5 toxicities due to SBRT from 2008 to 2022

	Grade 5 toxicity (accumulated SBRT cases until each survey year)						
	~2008	~2010	~2014	~2016	~2018	~2020	~2022
(a) Accumulated SBRT case number	3107	7938	15 073	19 493	27 453	38 476	56 686
Radiation pneumonitis	28	42	41	40	43	55	46
Hematochezia, lung hemorrhage	3	3	3	4	5	6	4
Liver damage	0	0	2	0	7	4	8
Radiation esophagitis	1	1	1	2	1	4	3
Digestive tube bleeding	0	0	0	3	3	4	5
Others	4	5	6	3	4	3	6
(b) Total	36	51	53	52	63	76	72
Total (100 × {(b)/(a)}, %)	1.2%	0.6%	0.4%	0.3%	0.2%	0.2%	0.1%

### Medical physicist/radiation technologist section—summary and general information for this survey

Table 4 presents the results from the medical physicist/radiation technologist questionnaire, with 405 responses (46.0% overall response rate) and 302 from DCCHs (65.5%). IMRT is implemented in 68.6% of facilities, planned in 5.9% and not planned in 25.4%, with the main barrier being a shortage of radiation oncologists (84.6%). SBRT is implemented in 76.3%, planned in 4.9% and not planned in 18.8%, with the same primary barrier (64.4%).

**Table 4:** Survey result of the questionnaire in the medical physicist/radiation technologist section (general information)

No.	Contents	*n*	Answer
4.1	Do you plan to implement IMRT?	405	Already: 278 (68.6%) Yes: 24 (5.9%) No: 103 (25.4%)
4.2	What are the reasons why IMRT cannot be implemented?	123	Shortage of radiation oncologists: 104 (84.6%) Shortage of medical physicists: 34 (27.6%) Shortage of radiological technologists: 29 (23.6%) Lack of education: 28 (22.8%) Lack of training environment: 18 (14.6%) No treatment machine for IMRT: 18 (14.6%) None in particular: 3 (2.4%) Lack of medical reimbursement: 3 (2.4%) No QA device: 1 (0.8%) Others: 5 (4.1%)
4.3	Do you plan to implement SBRT?	405	Already: 309 (76.3%) Yes: 20 (4.9%) No: 76 (18.8%)
4.4	What are the reasons why SBRT cannot be implemented (duplicate)?	90	Shortage of radiation oncologists: 58 (64.4%) Shortage of medical physicists: 30 (33.3%) Shortage of radiological technologists: 23 (25.6%) Lack of education: 25 (27.8%) Lack of training environment: 16 (17.8%) No treatment machine for SBRT: 15 (16.7%) None in particular: 6 (6.7%) Lack of medical reimbursement: 3 (3.3%) No respiratory motion management device: 2 (2.2%) No QA device: 1 (1.1%) Others: 7 (7.8%)
4.5	Did you conduct a dosimetric audit in the past 3 years?	405	Yes (ANTM): 280 (69.1%) Yes (Others): 75 (18.5%) No: 50 (12.3%)
4.6	Does your facility have an approved department for quality assurance and treatment planning? (e.g. radiation therapy quality assurance office, medical physics office, etc.)	405	Yes: 91 (22.5%) No: 314 (77.5%)
4.7	Number of radiology technologists, medical physicists, etc. in the radiotherapy department	2908	Full-time employment: 2717 (93.4%) Non-regular employment: 191 (6.6%)
4.8	Number of radiology technologists, medical physicists, etc. in the approved department for quality assurance and treatment planning	686	Full-time employment: 647 (94.3%) Non-regular employment: 39 (5.7%)
4.9	Number of radiology technologists, medical physicists, etc. in the approved department for quality assurance and treatment planning (excluding irradiation task)	383	Full-time employment: 342 (89.3%) Non-regular employment: 41 (10.7%)

Dosimetric audits are performed by 69.1% (Association for Nuclear Technology in Medicine (ANTM)), 18.5% (other) and not conducted by 12.3%. Only 22.5% of facilities have an approved department for quality assurance or medical physics. Among technologists and physicists in radiotherapy, 93.4% are full-time, while 94.3% are full-time in approved quality assurance and planning departments, with 89.3% dedicated specifically to those functions.

### Medical physicist/radiation technologist section—installation status of treatment machine

**Fig. 2:**
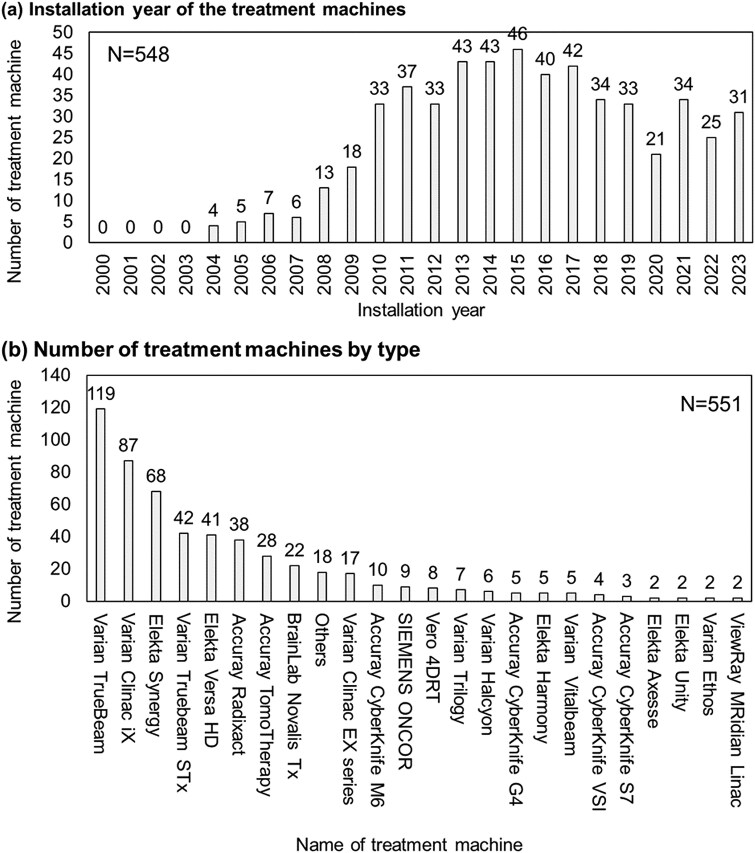
Survey results of the number of treatment machines. (A) The installation year of the treatment machines, and (B) the number of treatment machines by type.

**Fig. 3:**
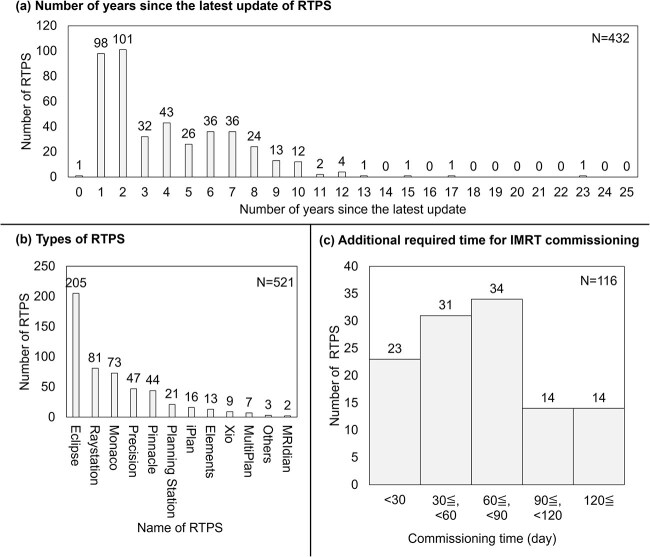
Survey results of the number of radiotherapy treatment planning system (RTPS)s. (A) The Number of years since the latest update of RTPS, (B) Types of RTPS, and (C) Additional required time for IMRT commissioning.

Figure 2 shows (i) The installation year of the treatment machines, and (ii) the number of treatment machines by type. The most common response was Varian TrueBeam, with the year of introduction peaking in 2015. Figure 3 also shows (i) the number of years since the latest update of RTPS, (ii) types of RTPS and (iii) additional required time for IMRT commissioning. The most common response was Eclipse, with the most common elapsed time since the most recent update being 2 years.

Table 5 summarizes the questionnaire results on treatment machines and devices in radiation therapy rooms. The most common IMRT and SBRT technique was ‘VMAT, Tomo helical’, used in 68.8% and 52% of cases, respectively. For image-guided devices in IMRT and SBRT, ‘kV-CBCT, MV-CBCT’ was the most frequently used (82.7%). Tumor position matching was the primary IGRT method (85.4%). The most common respiratory motion management device was ‘abdominal compression immobilization’ (43.8%), and 47.8% used ‘abdominal compression’ as a motion management method.

**Table 5:** Survey result of the questionnaire in the medical physicist/radiation technologist section (radiation therapy room information)

No.	Contents	n	Answer
5.1	IMRT technique (duplicate)	548	VMAT, TomoHelical: 377 (68.8%) Fixed IMRT (MLC, TomoDirect): 143 (26.1%) Not use any IMRT techniques: 95 (17.3%) Fixed IMRT (Circle, IRIS collimators): 20 (3.7%) Others: 1 (0.2%)
5.2	SBRT technique (duplicate)	548	VMAT, TomoHelical: 285 (52%) Fixed beam 3DCRT: 224 (40.9%) Not use any SBRT techniques: 92 (16.8%) Fixed IMRT (MLC, TomoDirect): 81 (14.8%) Conformal arc: 71 (13%) Combination with fixed beam 3DCRT and conformal arc: 35 (6.4%) Others: 2 (0.4%)
5.3	Image-guided devices used in IMRT and SBRT (as final approval) (duplicate)	548	kV-CBCT、MV-CBCT: 453 (82.7%) EPID: 119 (21.7%) Ceiling/floor mounted IGRT: 116 (21.2%) Gantry mounted IGRT: 103 (18.8%) SGRT system: 17 (3.1%) MV-portal: 16 (2.9%) CT-on rails: 12 (2.2%) Others: 10 (1.8%) MRI: 4 (0.7%)
5.4	IGRT methods used in IMRT and SBRT (as final approval). (duplicate)	548	Tumor position matching: 468 (85.4%) Bone position matching: 388 (70.8%) Surface position matching: 23 (4.2%) Others: 10 (1.8%)
5.5	Respiratory motion management devices used in IMRT and SBRT (duplicate)	548	Abdominal compression immobilization: 240 (43.8%) Varian RPM (RGSC): 178 (32.5%) Abches: 108 (19.7%) No devices: 46 (8.4%) SGRT system: 44 (8%) ANZAI(AZ-733VI): 28 (5.1%) Radixact Synchrony: 22 (4%) CyberKnife Synchrony: 21 (3.8%) Others: 14 (2.6%) Varian Triggered Imaging: 11 (2%) RT-RT (SyncTrax): 9 (1.6%) Vero 4DRT: 7 (1.3%) MRI: 3 (0.5%)
5.6	Respiratory motion management methods used in IMRT and SBRT (duplicate)	548	Abdominal compression: 262 (47.8%) Breath-hold: 219 (40%) Gating: 121 (22.1%) No devices: 52 (9.5%) Tracking: 48 (8.8%) Tracking (interception): 19 (3.5%) Others: 14 (2.6%)

Abbreviations: 3DCRT (three-dimensional conformal radiation therapy), EPID (electronic portal imaging device), SGRT (surface guided radiation therapy), MV-portal (megavoltage portal imaging), CT (computed tomography).

### Medical physicist/radiation technologist section—installation status of treatment planning system

Table 6 presents the questionnaire results on radiation treatment planning systems (RTPS). For IMRT commissioning, 29.8% reported it was introduced after 3DCRT commissioning. The most common additional time required for IMRT commissioning was 60–90 days. Commissioning support was utilized by 44.4% of facilities. Regarding vendor or external data usage, 19.3% said ‘Yes’, while 72.1% used standard measured data, and 8.5% used special measured data.

**Table 6:** Survey result of the questionnaire in the medical physicist/radiation technologist section (RTPS information)

No.	Contents	n	Answer
6.1	Was IMRT introduced at the same time as 3DCRT at start-up?	460	After 3DCRT commissioning: 137 (29.8%) At the same time of 3DCRT commissioning: 323 (70.2%)
6.2	Did you use any commissioning supports (vender, individual, organization, etc.) outside the facility?	482	Yes: 214 (44.4%) No: 268 (55.6%)
6.3	Did you use the beam data and beam model provided by the vender or other facility?	305	Yes: 59 (19.3%) No (standard measured data at own facility): 220 (72.1%) No (special measured data at own facility): 26 (8.5%)
6.4	Do you use knowledge-based or AI technologies for IMRT or SBRT treatment planning (excluding the script function)?	296	Yes (contouring and optimization): 8 (2.7%) Yes (optimization): 10 (3.4%) Yes (contouring): 39 (13.2%) No: 239 (80.7%)
6.5	Do you use the script function for IMRT or SBRT treatment planning?	295	Yes (contouring and optimization): 24 (8.1%) Yes (optimization): 12 (4.1%) Yes (contouring): 32 (10.8%) No: 227 (76.9%)

Abbreviations: AI (artificial intelligence).

For knowledge-based and AI technologies, 2.7% used them for both contouring and optimization, 3.4% for optimization and 13.2% for contouring. Similarly, for the script function, 8.1% used it for both, 4.1% for optimization and 10.8% for contouring.

### Medical physicist/radiation technologist section—treatment planning task

**Fig. 4:**
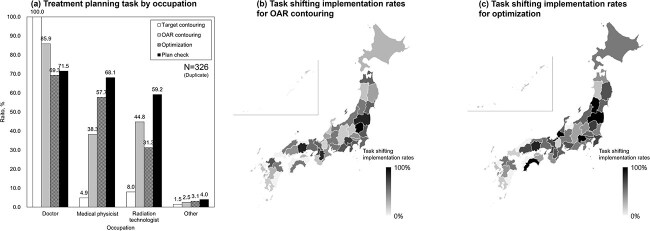
(A) Treatment planning tasks by occupation. White: target contouring; gray: organs at risks (OAR) contouring; shaded: optimization; black: plan checking for radiation oncologists, medical physicists, radiation technologists and others, respectively. (B) and (C) show regional comparative data on task shifting implementation in Japan. The task shifting rate for each task was calculated as the percentage of facilities responding to this study within each prefecture that reported a medical physicist or radiation technologist performing the respective task. (B) illustrates data for OAR contouring, while (C) presents data for optimization.

Figure 4A shows the tasks of treatment planning by occupation. Treatment planning tasks were mostly handled by radiation oncologists. On the other hand, 57.7% of the responding facilities, medical physicists performed treatment planning (Beam arrangement/Optimization), and 31.3% of the facilities, radiation technologists perform these tasks. Additionally, treatment plan checks were carried out by radiation oncologists and medical physicists in about 68.1% of the facilities, and by radiation technologists in 59.2% of the facilities.

We also included regional comparative data on task shifting implementation in Japan, presented in Fig. 4B and C. The task shifting rate for each task was calculated as the percentage of facilities responding to this study within each prefecture that reported a medical physicist or radiation technologist performing the respective task. These figures highlight variations in task shifting achievement rates across different prefectures.

### Medical physicist/radiation technologist section—treatment planning technique

Table 7 outlines the results regarding treatment planning techniques. For CT imaging methods in IMRT and SBRT planning, ‘4DCT’ was most common (51.5%). For dose calculation in cases with respiratory motion, ‘Slow Scan’ was the most frequent method (17.5%). For travel length measurement or ITV setting, ‘4DCT’ was predominant (77.6%).

**Table 7:** Survey result of the questionnaire in the medical physicist/radiation technologist section (treatment planning procedure information)

No.	Contents	n	Answer
7.1	CT Imaging method for IMRT and SBRT treatment planning (duplicate)	334	4DCT: 172 (51.5%) Free-breathing CT: 122 (36.5%) Exhale breath-hold CT: 109 (32.6%) Inhale breath-hold CT: 84 (25.1%) Fast scan CT: 75 (22.5%) Deep inspiration breath-hold CT: 31 (9.3%) Deep expiration breath-hold CT: 15 (4.5%) Others: 9 (2.7%)
7.2	CT images used for dose calculation in cases with respiratory motion during IMRT and SBRT treatment planning (Duplicate)	325	Slow scan CT: 57 (17.5%) 4DCT AveIP: 45 (13.8%) Free-breathing CT: 30 (9.2%) Exhale breath-hold CT: 29 (8.9%) Fast scan CT: 26 (8%) Inhale breath-hold CT: 22 (6.8%) Optimal 4DCT phase: 22 (6.8%) Deep inspiration breath-hold CT: 14 (4.3%) Deep expiration breath-hold CT: 8 (2.5%) Others: 5 (1.5%) 4DCT MIP: 4 (1.2%)
7.3	Images acquired for travel length measurement or ITV setting during IMRT or SBRT treatment planning (Duplicate)	317	4DCT: 246 (77.6%) Multiple phases of breath-hold CT: 127 (40.1%) Fluoroscopy: 38 (12%) Others: 25 (7.9%) MR cine: 5 (1.6%)
7.4	Main irradiation techniques used in SBRT treatment planning	323	VMAT: 152 (47.1%) 3DCRT: 108 (33.4%) Fixed IMRT: 31 (9.6%) Conformal arc: 14 (4.3%) Others: 12 (3.7%) 3DCRT+Conformal arc: 6 (1.9%)

Abbreviations: 4DCT (Four Dimensional Computed Tomography), AveIP (Average intensity projection)

**Fig. 5:**
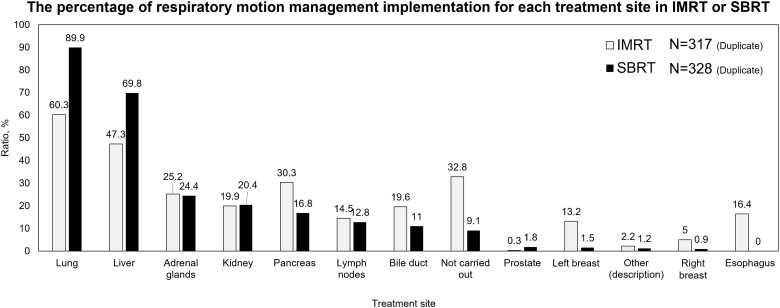
The percentage of respiratory motion management implementation for each treatment site in IMRT or SBRT.

Figure 5 shows respiratory motion management implementation by treatment site, with the lung being the most common—60.3% for IMRT and 89.9% for SBRT. The main SBRT methods were ‘VMAT’ (47.1%), followed by ‘3D-CRT’ (33.4%), ‘Fixed IMRT’ (9.6%), ‘Conformal arc’ (4.3%), ‘Other’ (3.7%) and ‘3DCRT + Conformal arc’ (1.9%). Table 8 highlights setup margins for different irradiation methods and cancer types, showing significant variation among facilities.

**Table 8:** Setup margins for each irradiation method for each treatment site from this survey

				Setup margin (mm)					
Treatment site	Irradiation technique	n		Anterior	Posterior	Left	Right	Superior	Inferior
Prostate cancer	IMRT	244	Minimum	1	0	1	1	1	1
			Median (IQR)	7 (5–8)	5 (4–8)	6 (5–8)	6 (5–8)	7 (5–8)	6 (5–8)
			Maximum	10	10	10	10	10	10
Prostate cancer	SBRT	37	Minimum	2	1	2	2	2	2
			Median (IQR)	5 (3–5)	3 (3–4)	5 (3–5)	5 (3–5)	5 (3–5)	5 (3–5)
			Maximum	7	5	6	6	7	6
Head and Neck cancer	IMRT	194	Minimum	1	1	1	1	1	1
			Median (IQR)	5 (5–5)	5 (5–5)	5 (5–5)	5 (5–5)	5 (5–5)	5 (5–5)
			Maximum	10	10	10	10	10	10
Lung cancer	IMRT	187	Minimum	1	1	1	1	1	1
			Median (IQR)	5 (5–5)	5 (5–5)	5 (5–5)	5 (5–5)	5 (5–5)	5 (5–5)
			Maximum	10	10	10	10	10	10
Lung cancer	SBRT	244	Minimum	1	1	1	1	1	1
			Median (IQR)	5 (4–5)	5 (4–5)	5 (4–5)	5 (4–5)	5 (4–5)	5 (4–5)
			Maximum	10	10	10	10	10	10
Spinal metastasis	SBRT	142	Minimum	0	0	0	0	0	0
			Median (IQR)	2 (2–3)	2 (2–3)	2 (2–3)	2 (2–3)	2 (2–3)	2 (2–3)
			Maximum	7	7	7	7	7	7

### Medical physicist/radiation technologist section—dose verification

Table 9 summarizes the questionnaire results on dose verification. The most common method for dose distribution verification was ‘3D-array (ArcCHECK Delta4)’ (81.7%). Fluence verification was ‘Not carried out’ by 69.8% of respondents, and 65.3% did not perform independent dose verification for IMRT. For differences between dose verification of regular IMRT and SBRT-IMRT, 75.1% reported using the ‘Same’ approach, while 17% used ‘Different tolerance or measurement methods’.

**Table 9:** Survey result of the questionnaire in the medical physicist/radiation technologist section (dose verification)

No.	Contents	n	Answer
9.1	Methods of dose distribution verification (duplicate)	289	3D-array (ArcCHECK, Delta4): 236 (81.7%) Film coronal: 86 (29.8%) Film sagittal: 69 (23.9%) Film axial: 42 (14.5%) 2d-array coronal: 38 (13.1%) 2d-array sagittal: 19 (6.6%) Others: 13 (4.5%) Not carried out: 8 (2.8%) 2d-array axial: 5 (1.7%)
9.2	Methods of fluence verification	288	Not carried out: 201 (69.8%) Treatment machine-supplied equipment measurements: 73 (25.3%) External equipment measurements: 14 (4.9%)
9.3	Methods of Independent dose verification	288	Not carried out: 188 (65.3%) Other RTPS dose calculation: 89 (30.8%) Independent calculation verification software other than RTPS: 11 (3.8%)
9.4	What is different about dose verification during SBRT and VMAT/IMRT compared to regular IMRT	289	Same: 217 (75.1%) Different tolerance or measurement methods: 49 (17%) Others: 23 (8%)
9.5	Do you perform dose verification during the treatment period?	304	Yes: 36 (11.8%) No: 259 (85.2%) Used to do it but not anymore: 9 (3%)
9.6	What type of dose verification during the treatment period do you perform (duplicate)?	36	Patient dose reconstruction from log-file: 13 (36.1%) Patient dose reconstruction from EPID fluence: 12 (33.3%) Patient dose reconstruction from Gantry-mounted transmission detector: 3 (8.3%) Fluence comparison (EPID vs. RTPS): 12 (33.3%) Fluence comparison (Log-file vs. RTPS): 9 (25%) Others: 9 (25%)
9.7	Who is in charge for dose verification during the treatment period (duplicate)?	36	Medical physicist: 24 (66.7%) Radiation technologist: 22 (61.1%) Radiation oncologist: 1 (2.8%) Others: 2 (5.6%)
9.8	The reason for not performing dose verification during the treatment (duplicate).	268	No equipment: 164 (61.2%) Shortage of working time: 107 (39.9%) Shortage of human resource: 87 (32.5%) No health insurance fee: 65 (24.3%) Others: 25 (9.3%) Was implemented but considered no longer necessary: 7 (2.6%)
9.9	If the issues in upper question are resolved, would you like to implement the dose verification during the treatment?	268	Eager to implement: 91 (34%) Somewhat open to implementing: 124 (46.3%) Somewhat resistant to implementing: 36 (13.4%) Opposed to implementing: 17 (6.3%)
9.10	Do you think dose verification during the treatment period is necessary?	300	Necessary: 87 (29%) Somewhat necessary: 166 (55.3%) Somewhat unnecessary: 36 (12%) Unnecessary: 11 (3.7%)

Abbreviations: RTPS (Radiotherapy treatment planning system)

During-treatment dose verification was conducted by 11.8% of respondents, with ‘Patient dose reconstruction from log-file’ (36.1%) and ‘Patient dose reconstruction from EPID fluence’ (33.3%) being common methods. In 66.7% of facilities, medical physicists handled dose verification. The main barrier to performing dose verification was ‘No equipment’ (61.2%). If barriers were resolved, approximately 80% were willing to implement dose verification. Over 80% considered dose verification during the treatment period ‘Necessary’ or ‘Somewhat necessary’.

### Medical physicist/radiation technologist section—other technical aspects and work environment related to IMRT/SBRT

Table 10 summarizes the questionnaire results on other techniques and the working environment. For primary IGRT matching, 97% of respondents indicated it is performed by radiation technologists, while 3% said it is performed by radiation oncologists. Rectal gas removal was most commonly performed by ‘nurses’ (69%), followed by ‘radiation oncologists’ (23.4%) and ‘radiology technicians’ (14.6%). Regarding hospital rules for rectal gas removal, 50.5% reported having ‘nothing’, 38.6% indicated ‘specific occupation performing’, 4.9% required ‘prior training’ and 0.9% used an ‘in-house certification system’.

**Table 10:** Survey result of the questionnaire in the medical physicist/radiation technologist section (Other techniques and working environment)

No.	Contents	*n*	Answer
10.1	Do the radiation technologists perform IGRT position matching? (primary matching)	333	Yes: 323 (97%) No: 10 (3%)
10.2	Who performs rectal gas removal in pelvic irradiation (duplicate)?	329	Nurse: 227 (69%) Radiation oncologist: 77 (23.4%) Radiation technologist: 48 (14.6%)
10.3	Are there any hospital rules about rectal gas removal (duplicate)?	329	Nothing: 166 (50.5%) Specific occupation performing: 127 (38.6%) Prior training: 16 (4.9%) In-house certification system: 3 (0.9%)
10.4	Have you been instructed by your superiors to reduce overtime work in the radiotherapy department?	331	Directed to do so, and overtime hours have decreased: 86 (26%) Directed to do so, but overtime hours remain unchanged: 107 (32.3%) Directed to do so, and overtime hours have increased: 9 (2.7%) Not directed to do so, but overtime hours have decreased: 23 (6.9%) Not directed to do so, but overtime hours remain unchanged: 100 (30.2%) Not directed to do so, but overtime hours have increased: 6 (1.8%)
10.5	Has the amount of time devoted to staff training within the radiotherapy department decreased?	333	Increased significantly: 3 (0.9%) Increased: 10 (3%) Unchanged: 215 (64.6%) Decreased: 84 (25.2%) Decreased significantly: 21 (6.3%)
10.6	Do you have enough training time during working hours (excluding overtime) to devote to education?	333	Can secure enough: 10 (3%) Can secure: 88 (26.4%) Unknown: 110 (33%) Cannot secure: 112 (33.6%) Cannot secure at all: 13 (3.9%)

**Fig. 6:**
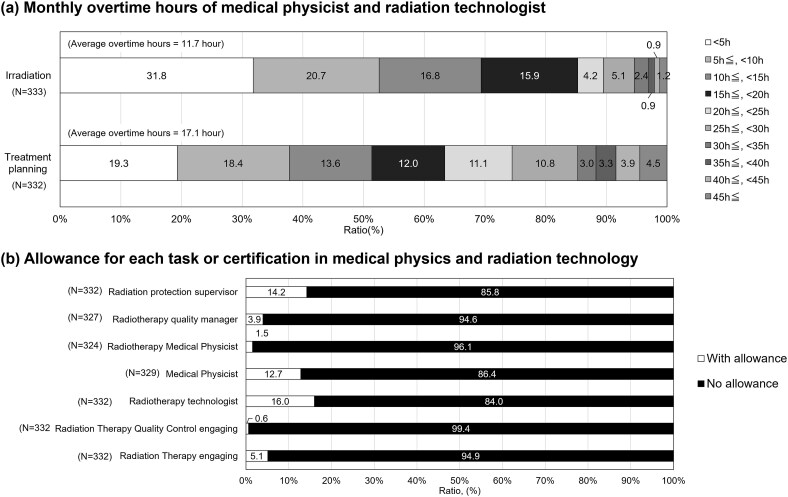
(A) Monthly overtime hours of medical physicist and radiation technologist. (B) Allowance for each task or certification in medical physics and radiation technology.

Over half of respondents reported being instructed to reduce overtime. Few facilities indicated an increase in educational time for staff, and less than 30% provided training during working hours. Figure 6 illustrates (i) monthly overtime hours for medical physicists and radiation technologists and (ii) allowances for tasks or certifications. The most common overtime was under 5 hours for both irradiation and planning. The average overtime hours were 11.7 for irradiation work and 17.1 for planning work.

### Medical physicist/radiation technologist section—classification of incidents for IMRT and SBRT

Table 11 shows the number of task classifications of incidents. The most common task classifications of incidents were ‘patient setup/pre-treatment’, ‘treatment planning/optimization’ and ‘patient positioning/immobilization and skin markers’, in that order. Similarly, Table 12 shows the number of cause classifications of incidents. The most common cause classifications of incidents were ‘carelessness’, ‘insufficient experience, knowledge and understanding’, and ‘communication errors between staff’, in that order.

**Table 11:** The number of task classifications of incidents regarding to IMRT and SBRT

Task classification of incident	n
Patient set-up/pre-treatment	78
Treatment planning/optimization	76
Patient positioning, immobilization and skin markers	76
Others	70
Position matching/IGRT	49
Imaging for treatment planning (CT, others)	44
Data transfer	43
Treatment plan validation (plan check, dose verification)	36
Irradiation	35
Target contouring	25
OAR contouring	19
Respiratory motion management	13
Commissioning	4

**Table 12:** The number of cause classifications of incidents regarding to IMRT and SBRT

Cause classification of incident	*n*
Carelessness	170
Insufficient experience, knowledge and understanding	112
Communication errors between staff	86
Deviation from the rules	62
Fatigue due to busyness, etc.	51
Misreading or recognition errors	45
Malfunction of equipment, software or facilities	35
Patient factors	24
Incorrect procedures/rules themselves	20
Others	15
Work environment	14
Natural disaster	1

## DISCUSSION

Although this study was conducted in Japan, the findings regarding the IMRT/SBRT technologies employed and the prescribed doses have the potential to contribute to the standardization of technologies and treatment protocols in other countries, thereby promoting international harmonization.

Regarding the results of the radiation oncologist section, the case of IMRT showed a flat trend in recent year. However, Fig. 1A shows that the number of IMRT cases is increasing for certain diseases, such as lung cancer. This increase may be due to past clinical trials [15, 16] demonstrating the effectiveness of IMRT, as well as the attention given to chemoradiotherapy combined with durvalumab from the results of the PACIFIC trial [17–19]. On the other hand, the case of SBRT showed an increasing trend. Especially, Fig. 1B shows a sharp rise in the number of cases involving spinal metastasis, prostate cancer and oligometastasis. This increase may be attributed to the fact that each of these conditions has been covered by insurance in Japan since 2020.

The top three reasons for not being able to implement IMRT were lack of human resources for radiation oncologists and technicians. Reliable data on the implementation rates of IMRT and SBRT in other countries are very limited. However, in recent years, a structural survey conducted by the Federation of Asian Organizations for Radiation Oncology found that Japan has an above-average implementation rate for SBRT and SRS but a below-average rate for IMRT [20]. In Japan, unlike in other countries, it is required that two radiation oncologists be present in the radiotherapy department specifically to perform IMRT. However, in this questionary, there were many requests for relaxation of the requirement of two radiation oncologists, so it is necessary to continue to discuss revising facility requirements with all relevant academic societies, government ministries and agencies. Tele-radiotherapy planning has also been investigated [21], and since some of the results in this study showed promise for use in IMRT, we believe that further investigation should continue as an option for equalization of IMRT.

Specific surveys on online ART implementation rates remain limited. The POP-ART RT survey revealed that 177 institutions from 40 countries completed the ART-related questions, with only 31% implementing either online or offline protocols [22]. Although guidelines for on-line adaptive radiation therapy (ART) have recently been published in Japan [23], only 4.2% (15 facilities) responded in this survey that they are implementing such therapy. However, further development is expected in the future, as information sharing has been conducted with a limited number of users, such as the first survey of online adaptive radiotherapy in Japan [24]. There was significant variation among facilities in the prescribed dose and method of prescribing in SBRT. There are also reports that prescribing methods vary even in foreign countries [25–27], and uniformity in SBRT prescribing doses remains an issue for the future. Grade 5 adverse events and their frequency in SBRT have also not changed significantly in recent years.

In recent years, various surveys have been conducted on the workload and educational environment of the radiotherapy technology and medical physics specialists [3, 4]. For the physicist / technologist section in this study, a more detailed rate of use of technologies related to high-precision radiation therapy was investigated. Especially, we were able to ascertain the implementation rate of treatment planning work (target/OAR contouring, optimization and plan check) by job type and the utilization rate of the latest technologies (start-up support, AI) for the first time in Japan. Since AI has a variety of uses in the field of radiation therapy [28–30], we believe that continued research on utilization rates is necessary. VMAT is becoming the predominant technology used for SBRT, which is consistent with the trend from previous reports [31, 32]. The setup margins for high-precision irradiation at each facility were also investigated to determine the variation and median values. Such a survey has never been done before and will be important data for further standardization. On the other hand, margin sizes have not changed much compared to past survey results for prostate cancer IMRT and lung SBRT [33, 34], suggesting that many institutions remain cautious about reducing margins. It was also clear that primary matching of IGRT by radiation technologists is actively practiced at many facilities in the current situation. On the other hand, for IMRT treatment planning, it is clear that there is still a heavy radiation oncologist workload, although there has been more task shifting compared to past reports [35], especially in optimization and beam setting. Regarding task shifting in treatment planning, for example, Dosimetrists are active as full-time professional planners specializing in treatment planning in the United States [36]. On the other hand, in the United Kingdom and many other countries in Europe, such a system has not been established [37]. In Japan, medical physicists and radiation technologists play an important role in replacing dosimetrists, and it is necessary to further develop a system unique to this country that utilizes them in the future. To ensure effective IMRT treatment planning, it is necessary to prepare educational opportunities. However, it is clear that educational opportunities are decreasing in many facilities, and allowances for the specialized professions required for high-precision irradiation are not being considered. The entire association needs to continue to seek solutions to these issues.

Some limitations of this study include the following: First, the number of responses declined from the previous survey, which may have affected the results. The reasons for the decrease in the response rate include respondent factors (e.g. lack of time to complete the survey due to work schedule changes, radiation oncologists no longer being dispatched, radiation therapy equipment (IMRT and SBRT) being discontinued, and respondents not feeling the need to respond), the method of notification (email-based, with only one mailing at the beginning of the survey), and the number of questions (the number of questions is large and burdensome to respondents). In recent years, there has been a report based on data from the Ministry of Health, Labor and Welfare [38,], and the results of this study should be interpreted with reference to such reports. In particular, the number of cases shown in Fig. 1 is not easy to interpret because the number of responding facilities varies from year to year. Second, the questionnaire was complex, and it was possible that the responding facilities were those with staff who had a deep knowledge of radiotherapy, while those that did not respond may lack such staff. In other words, responses from facilities that did not provide high-precision radiotherapy treatment may be insufficient. Third, the data such as GammaKnife and CyberKnife operated by the Department of Neurosurgery were missing. Third, the survey focused solely on the use of IMRT and SBRT without assessing the clinical outcomes associated with these techniques. Future efforts should consider conducting a large-scale national survey to evaluate the outcomes of patients treated with IMRT and SBRT. We plan to continue conducting such surveys every two years to identify trends in technological advancements for IMRT/SBRT in Japan.

## CONCLUSION

The report clarified the actual situation in Japan regarding the use of IMRT/SBRT up to 2023. According to the Nationwide survey, IMRT and SBRT are getting popular, however, the facility requirements which mandate the presence of at least two radiation oncologists prevents IMRT from becoming more widespread in Japan. Revising facility requirements and applying new technologies such as AI and remote radiotherapy planning are expected for the further widespread adoption of high-precision radiotherapy.
